# *Fusarium* head blight incidence and mycotoxin accumulation in three durum wheat cultivars in relation to sowing date and density

**DOI:** 10.1007/s00114-017-1528-7

**Published:** 2017-12-05

**Authors:** Anna Gorczyca, Andrzej Oleksy, Dorota Gala-Czekaj, Monika Urbaniak, Magdalena Laskowska, Agnieszka Waśkiewicz, Łukasz Stępień

**Affiliations:** 1 Department of Agricultural Environment Protection, Agricultural University in Kraków, Mickiewicza 21, 31-120 Kraków, Poland; 2 Institute of Plant Production, Agricultural University in Kraków, Mickiewicza 21, 31-120 Kraków, Poland; 3 Department of Agrotechnology and Agricultural Ecology, Agricultural University in Kraków, Mickiewicza 21, 31-120 Kraków, Poland; 4 Department of Pathogen Genetics and Plant Resistance, Institute of Plant Genetics, Polish Academy of Sciences, Strzeszyńska 34, 60-479 Poznań, Poland; 5 Department of Chemistry, Poznań University of Life Sciences, Wojska Polskiego 75, 60-625 Poznań, Poland

**Keywords:** Deoxynivalenol, Durum wheat, *Fusarium*, Moniliformin, Nivalenol, Zearalenone

## Abstract

Durum wheat (*Triticum turgidum* var. *durum*) is an important crop in Europe, particularly in the Mediterranean countries. Fusarium head blight (FHB) is considered as one of the most damaging diseases, resulting in yield and quality reduction as well as contamination of grain with mycotoxins. Three winter durum wheat cultivars originating from Austria, Slovakia, and Poland were analyzed during 2012–2014 seasons for FHB incidence and *Fusarium* mycotoxin accumulation in harvested grain. Moreover, the effects of sowing density and delayed sowing date were evaluated in the climatic conditions of Southern Poland. Low disease severity was observed in 2011/2012 in all durum wheat cultivars analyzed, and high FHB occurrence was recorded in 2012/2013 and 2013/2014 seasons. *Fusarium graminearum* was the most abundant pathogen, followed by *Fusarium avenaceum*. Through all three seasons, cultivar Komnata was the most susceptible to FHB and to mycotoxin accumulation, while cultivars Auradur and IS Pentadur showed less symptoms. High susceptibility of cv. Komnata was reflected by the number of *Fusarium* isolates and elevated mycotoxin (deoxynivalenol, zearalenone, and moniliformin) content in the grain of this cultivar across all three seasons. Nivalenol was identified in the samples of cv. Komnata only. Genotype-dependent differences in FHB susceptibility were observed for the plants sown at optimal date but not at delayed sowing date. It can be hypothesized that cultivars bred in Austria and Slovakia show less susceptibility towards FHB than the cultivar from Poland because of the environmental conditions allowing for more efficient selection of breeding materials.

**Electronic supplementary material:**

The online version of this article (10.1007/s00114-017-1528-7) contains supplementary material, which is available to authorized users.

## Introduction

Durum wheat (*Triticum turgidum* var. *durum*) is an important crop in EU member countries, where it is cultivated on the area of nearly 3 mln ha, and according to the European Commission reports (2014), the top yields are between 5 and 6 t ha^−1^. The main areas of cultivation include four traditional regions—Italy, Greece, Spain, and France. Outside the Mediterranean area, lower productivities are recorded and breeders’ efforts are focused on improving the yield-forming potential as well as crop quality. Important features of wheat cultivar evaluation are drought tolerance, resilience to low temperatures, and other environmental stresses, as well as considerable level of resistance to diseases and pests (Garcia del Moral et al. 2003; Labuschagne et al. 2009; Royo et al. 2004, 2006, 2014). New durum wheat cultivars have thermal requirements similar to common wheat; however, the reproductive phase should proceed at higher temperatures (Labuschagne et al. 2009). The xerophytic character of durum wheats results in their relatively high resistance to water deficit. Water stress tolerance is the result of the cumulative action of various characteristics and physiological processes (Janeczko et al. 2016). The lack of suitable crop rotation favors plant infection with fungal pathogens, mainly belonging to the *Fusarium* genus. This multi-species complex is responsible for a number of diseases of small grain cereals, with Fusarium head blight (FHB) being the most damaging. Besides decreasing the grain quality and yield, it results in massive accumulation of mycotoxins with deoxynivalenol (DON) and its derivatives as prevailing metabolites, followed by zearalenone (ZON) and moniliformin (MON) (Covarelli et al. 2015; Wiśniewska et al. 2014).

Durum wheat requires chemical protection, especially in humid areas (Hossard et al. 2014). Such practices increase grain yield, decrease the infection of vegetative parts and heads, and, finally, lower the mycotoxin contamination. Although, the anti-fungal spraying delays plant aging, no significant influence of plant protection practices on the technological quality of durum wheat grain has been reported (Abad et al. 2004; Blandino et al. 2009; Gana et al. 2011; Lori et al. 2003). Optimum conditions seem to be crucial factors in the performance of durum wheat, as plant vigor and severity of the diseases are also determined by sowing density and time. Nevertheless, genetic background plays an important role in plant development, particularly in terms of resistance to diseases and contamination of grain with mycotoxins.

The main scientific aims of the study were (i) to evaluate the effect of three sowing densities and two sowing dates on the FHB incidence and severity on three winter durum wheat cultivars of different origin, (ii) to assess the accumulation of the most important *Fusarium* mycotoxins in the small grain cereals in the climatic conditions of the Southern Poland, and, finally, (iii) to identify *Fusarium* species present in the infected heads.

## Materials and methods

### Plant cultivation conditions

The field experiments (growing seasons: 2011/2012, 2012/2013, and 2013/2014) were conducted near Kraków (Southern Poland, 50° 06′ 52″ N; 20° 04′ 23″ E) in randomized block design, plots of 10 m^2^ each, with three replications.

Experimental factors were

- Three cultivars of winter durum wheat: Komnata (Poland), Auradur (Austria), and IS Pentadur (Slovakia).
- Sowing dates—optimum (25–30 September) and delayed (15–20 October).
- Sowing densities—400, 500, and 600 germinated seeds on square meter.

The pre-crop was potato or oilseed rape. After harvesting the previous crop, full soil tillage was performed. A standard chemical protection was applied according to the general recommendations, i.e., seed treatment, herbicide (Lintur 70 WG 150 g ha^−1^; active ingredients triasulfuron and dicamba), fungicides (Tilt Turbo 575 EC 1 L ha^−1^ at tillering phase and Tilt Turbo 575 EC 0.6 L ha^−1^ with Amistar 250 SC 0.6 L ha^−1^ at heading phase; active ingredients propiconasol, fenpropidin, and azoxystrobin, respectively), and a growth regulator (Moddus 250 EC 0.4 L ha^−1^ at heading phase; active ingredient trinexapac-ethyl).

Mineral fertilizers applied were

- Granular triple superphosphate 40% P_2_O_5_ 80 kg ha^−1^ P_2_O_5_ before sowing.
- Potassium salt 60% K_2_O 150 kg ha^−1^ K_2_O before sowing.
- Ammonium nitrate 34% N in three doses (first 80 kg ha^−1^ at tillering phase, second 40 kg ha^−1^ at stem elongation phase, and third 40 kg ha^−1^ at heading phase).

**Fig. 1:**
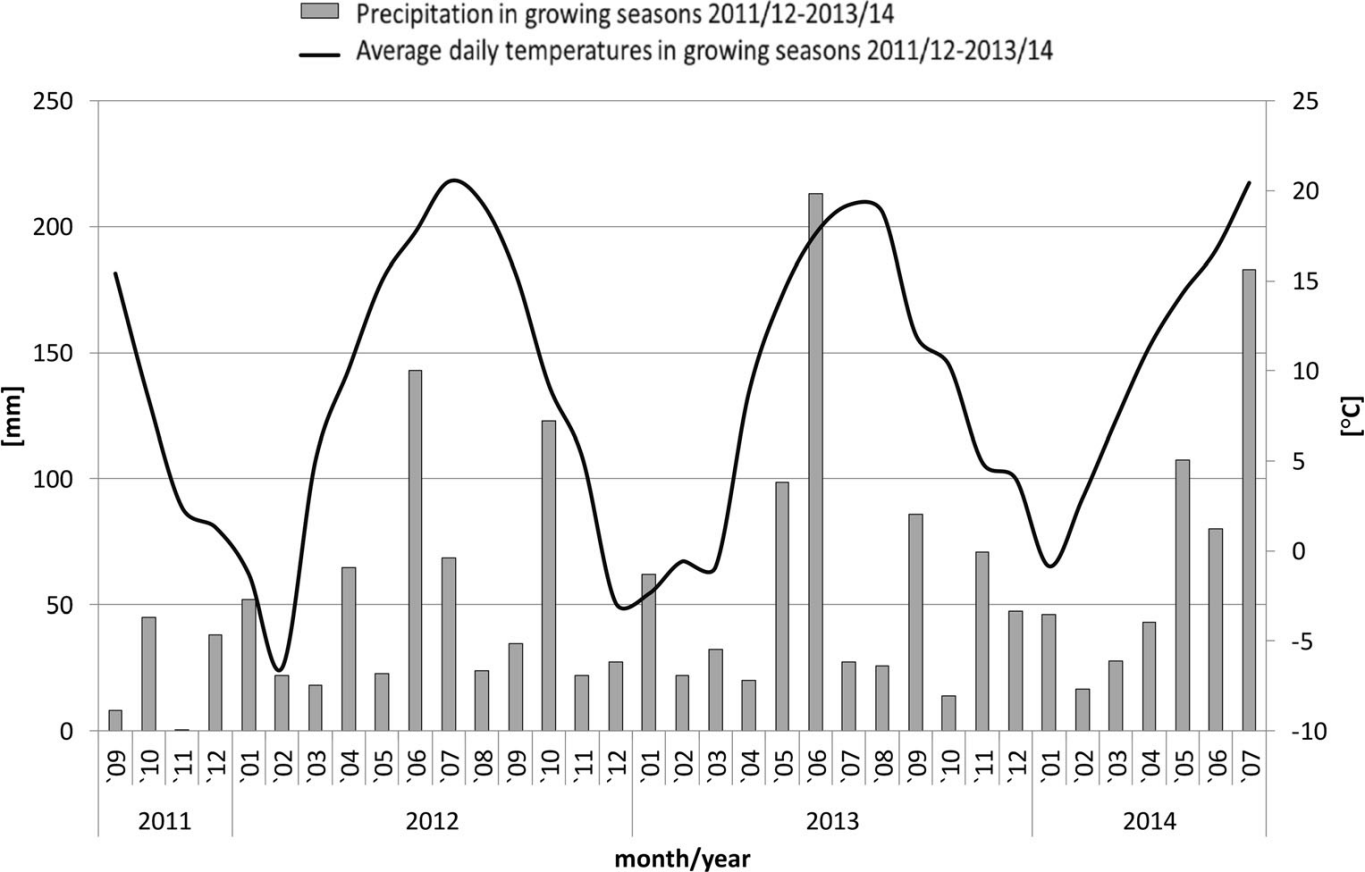
Weather conditions of the research area observed in the growing seasons (average temperatures [°C] and total precipitation [mm] for each month of the 3-year study (September 2011–July 2014)

Weather conditions were monitored by Advance Automatic Weather Station System WS-GP2 (Delta-T Devices, Cambridge, UK) located near the field experiments. The three seasons’ data on monthly average temperatures and total precipitation are presented on Fig. 1.

### FHB severity assessment and disease index calculation

Evaluation of FHB infection was performed in the grain maturation phase in 8° scale where 1° = healthy heads, 2° ≤ 15%, 3° = 15–30%, 4° = 30–45%, 5° = 45–60%, 6° = 60–75%, 7° = 75–90%, and 8° = 90–100% area of the head with disease symptoms. All diseased heads per plot were recorded to evaluate the FHB incidence. The evaluation scale was converted to a disease index (DI) factor according to the formula proposed by Pierre and Regnault (1982):

$$ \mathrm{DI}=\frac{\sum_{i=2}^7\left[2\left(i-2\right)+1\right]{n}_i}{\sum_{i=1}^7{n}_i} $$

where *n*_*i*_ denotes the number of plants within the category *i* (each of the evaluation groups).

### Fusarium strain isolation

Durum wheat cultivars were harvested at full plant maturity. Diseased heads were randomly chosen for pathogen isolation and identification, regardless of the DI recorded for the plot or cultivar. Grains from diseased heads exposed to natural infection by *Fusarium* fungi (one kernel per head, three heads per plot) were plated aseptically on the potato dextrose agar (PDA, Oxoid, Basingstoke, UK) medium and cultured for 5–7 days at 20–25 °C and 12-h photoperiod in triplicate. Multiple species infecting the same head were observed frequently; they were all isolated independently. Specifically, more than one *Fusarium* species could be isolated from a single kernel. Other fungal genera (e.g., *Epicoccum*, *Microdochium*, *Alternaria*) were also present (results not shown).

Individual *Fusarium* strains were isolated using Leslie and Summerell manual (Leslie and Summerell 2006) and maintained in pure cultures for 7 days on PDA medium for genomic DNA extraction. All isolates of *Fusarium* species from wheat heads were deposited in the Plant Pathogenic Fungal Strain Collection of the Institute of Plant Genetics, Polish Academy of Sciences, Poznań, Poland.

### DNA extraction, molecular species, and chemotype identification

Genomic DNA was extracted using a modified CTAB (hexadecyltrimethylammonium bromide) method described earlier (Stępień et al. 2004). The concentrations of DNA extracts were quantified using Nanodrop® spectrophotometer and stored at − 20 °C. Three *Fusarium* species-specific markers were used: Fc01 marker (amplicon of 570 bp) to identify *F. culmorum*, Fg16 marker (282 bp) specific for *F. graminearum*, and Fa marker (900 bp) to determine *F. avenaceum* (Chełkowski et al. 2012). The complete list of primers used is presented in Table 1. The isolates of other species were species identified on the basis of the sequence analysis of a variable fragment of the *translation elongation factor 1α* gene (*tef-*1α) as described by Stępień et al. (2016). The TRI7 (625 bp) marker was used to identify the NIV chemotype (Table 1). PCRs were done in 20 μL aliquots using C-1000 thermal cyclers (BioRad, Hercules, CA, USA). Each reaction contained 0.4 μL of Phire II HotStart Taq DNA polymerase (Thermo Scientific, Espoo, Finland), 4 μL of 5× PCR buffer, 12.5 pmol of forward/reverse primers, 2.5 mM of each dNTP, and about 20 ng of fungal DNA. PCR conditions were as follows: 30 s at 98 °C; 35 cycles of 5 s at 98 °C, 5 s at 63 °C, and 15 s at 72 °C; and 1 min at 72 °C. Amplicons were electrophoresed in 1.5% agarose gels (Invitrogen) with 2% GELRED dye (Biotium).

**Table 1:** PCR primers used for species-specific marker and NIV chemotype identification, target species/gene, and sequence

Primer designation	Target gene/species	5′ > 3′ sequence
FaF	*F. avenaceum*	AGCATTGTCGCCACTCTC
FaR		GTTTGGCTCTACCGGGACTG
Fc01F	*F. culmorum*	ATGGTGAACTCGTCGTGGC
Fc01R		CCCTTCTTACGCCAATCTCG
Fg16F	*F. graminearum*	CTCCGGATATGTTGCGTCAA
Fg16R		GGTAGGTATCCGACATGGCAA
Ef 728 M	Translation elongation factor 1α (*tef*-1α)	CATCGAGAAGTTCGAGAAGG
Tef1R		GCCATCCTTGGAGATACCAGC
Tri7F	Nivalenol (NIV) chemotype	ATCGTGTACAAGGTTTACG
Tri7NIV		TTCAAGTAACGTTCGACAAT

PCR-amplified fragments were purified with exonuclease I (Thermo Scientific) and FastAP alkaline phosphatase (Thermo Scientific) using the following program: 30 min at 37 °C and 15 min at 80 °C. Both DNA strands were labeled according to Stępień et al. (2012) using the same primers (Table 1) and the BigDyeTerminator 3.1 kit (Applied Biosystems, Foster City, CA, USA) and subsequently precipitated with 96% ethanol. Sequence reading was performed using Applied Biosystems equipment. Sequences were aligned using BLASTn algorithm to the GenBank-deposited reference strain sequences of individual *Fusarium* species.

### Mycotoxin analysis

#### Standards and chemical reagents

ZON, deoxynivalenol (DON), nivalenol (NIV), and MON standards were purchased with a standard grade certificate from Sigma-Aldrich (Steinheim, Germany). Organic solvents (HPLC grade) and all the other chemicals were also purchased from Sigma-Aldrich (Steinheim, Germany). Water for the HPLC mobile phase was purified using a Milli-Q system (Millipore, Bedford, MA, USA).

#### Extraction and purification procedure

Ten grams of ground kernels were subjected to mycotoxin extraction as described previously (Tomczak et al. 2002; Wiśniewska et al. 2014). The eluates were evaporated to dryness at 40 °C under a stream of nitrogen, and the dry residue was stored at − 20 °C until HPLC analyses.

#### HPLC analysis

The chromatographic system consisted of Waters 2695 high-performance liquid chromatography unit (Waters, Milford, USA) coupled with (i) Waters 2996 Photodiode Array Detector with Nova Pak C-18 column (300 × 3.9 mm) for DON and NIV (*λ* = 224 nm) and MON *(λ* = 229 nm) analysis and (ii) Waters 2475 Multi λ Fluorescence Detector (*λ*_EX_ = 274 nm, *λ*_EM_ = 440 nm) and Waters 2996 Photodiode Array Detector with Nova Pak C-18 column (150 × 3.9 mm) for ZON analysis. Mycotoxins were re-dissolved and separated according to Wiśniewska et al. (2014). Quantification of mycotoxins was performed by measuring the peak areas at the retention time according to relevant calibration curve. Limits of detection were 0.5 ng g^−1^ for ZON, 10 ng g^−1^ for DON and NIV, and 5 ng g^−1^ for MON.

### Statistical analyses

Data regarding DI were analyzed by three-way analysis of variance (ANOVA). Graphs were plotted using the means and standard errors (SE) for each data point. A post hoc comparison was conducted using Tukey’s multiple range test (*P* = 0.05). All calculations were carried out using the STATISTICA 10.0 (StatSoft, Inc., USA) software package.

## Results

### Weather conditions

Weather conditions (mean temperatures and precipitation) throughout the three seasons of the study were monitored and summarized (Fig. 1). The 2011/2012 season was dry with low rainfall during emergence and spring resuming of vegetation (March–May). In addition, the temperatures of the 2011/2012 season were slightly higher compared to the long-term data, but the averages for January and February were lower than in subsequent seasons. The season 2012/2013 brought the highest precipitation in June and the lowest in July (Fig. 1). The precipitation in the 2013/2014 season was significantly higher than recorded in the area for the long-term data, particularly during spring and summer (May–July).

### FHB assessment

**Fig. 2:**
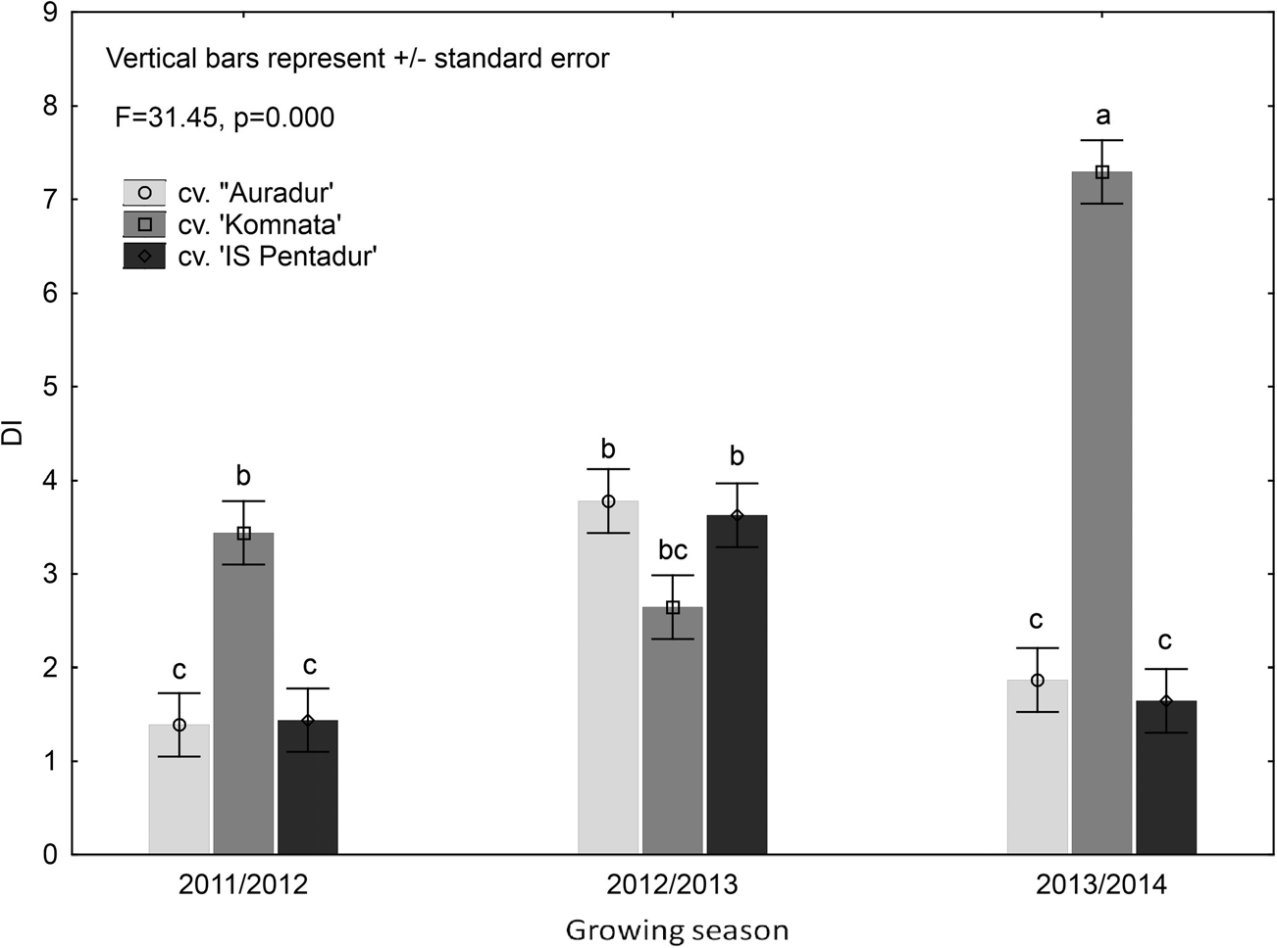
Mean *Fusarium* head blight (FHB) severity (DI (disease index)) on the three durum wheat cultivars used during this 3-year study (2012–2014)

DI was measured independently for each cultivar in each season. Significant variance was observed in FHB incidence during this 3-year study among the three cultivars tested (Fig. 2). In general, significantly more FHB symptoms were observed on plants during 2012/2013 and 2013/2014 seasons than in 2011/2012. Statistical significance of the factor combinations studied during the three seasons is shown in Table 3. In 2011/2012 and 2013/2014, cv. Komnata exhibited the highest infection symptoms, while in season 2012/2013, it was the least diseased cultivar. Cultivars Auradur and IS Pentadur displayed low FHB indices in 2011/2012 and 2013/2014, but significantly higher in 2012/2013 (Fig. 2).

### Fusarium species identification

**Fig. 3:**
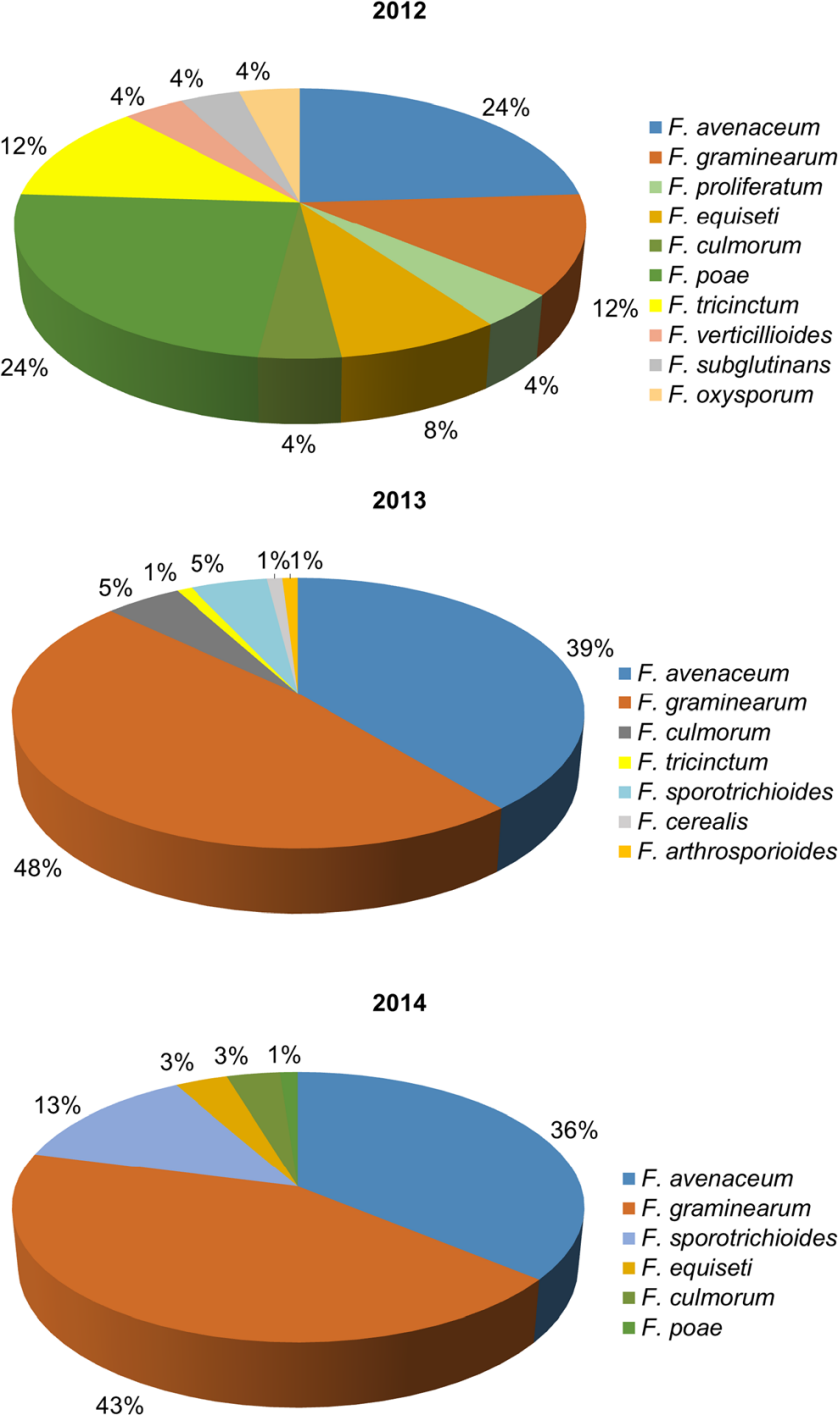
Frequencies of all *Fusarium* species isolated in 2011/2012–2013/2014 seasons from the grain of the three durum wheat cultivars grown in Southern Poland

Low *Fusarium* spp. abundance was observed in the 2011/2012 in all durum wheat cultivars analyzed (Table 2), reflected by a low number of *Fusarium* pathogens isolated from the grain. However, no significant differences between cultivars were recorded among all three seasons. The greatest species variance was found in the 2011/2012, though the number of isolates obtained was lower compared to the 2012/2013 and 2013/2014 (Fig. 3).

**Table 2:** Total number of *Fusarium* isolates obtained from three durum wheat cultivars tested across the 3-year survey

Cultivar	Total number of *Fusarium* isolates in season			Total
	2011/2012	2012/2013	2013/2014	
Auradur	6	31	31	68
Komnata	10	32	30	72
IS Pentadur	9	37	25	71
Total	25	100	86	211

The abundance of individual FHB-related species varied among seasons, particularly in the 2011/2012, when lower number of isolates was observed. Some of the species identified were exclusive for this season, e.g., *Fusarium subglutinans*, *Fusarium proliferatum*, and *Fusarium verticillioides*. *Fusarium* species composition of the natural pathogen populations in the 2012/2013 and 2013/2014 were roughly similar (Fig. 3). In the 2012/2013 and 2013/2014 seasons, when high FHB incidence was recorded, *F. graminearum* was the most abundant pathogen, followed by *F. avenaceum*. Moreover, *F. avenaceum* was also found at the highest frequency in the 2011/2012 season, when the FHB incidence was low (Fig. 3). No specific correlations between *Fusarium* species and wheat cultivars were observed (results not shown).

### Sowing dates and densities

**Fig. 4:**
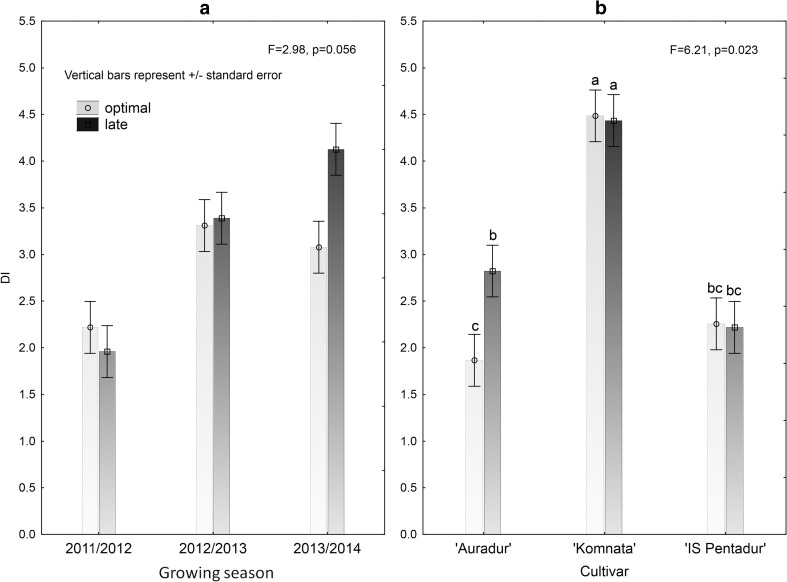
Effects of delayed vs. optimal sowing dates on overall FHB incidence during 2011/2012–2013/2014 seasons (**a**) in the three durum wheat cultivars studied and individual cultivar reactions (**b**). The differences were statistically significant only when the cultivars were compared

Two different sowing dates were analyzed: optimal and delayed (3 weeks after optimal sowing date). In the 2013/2014 season, an increase of disease symptoms was observed for the delayed sowing date (Fig. 4). Interestingly, when the influence of delayed sowing date on individual cultivars was compared, only cv. Auradur showed more FHB symptoms for the delayed sowing date than for the optimal date (Fig. 4).

**Fig. 5:**
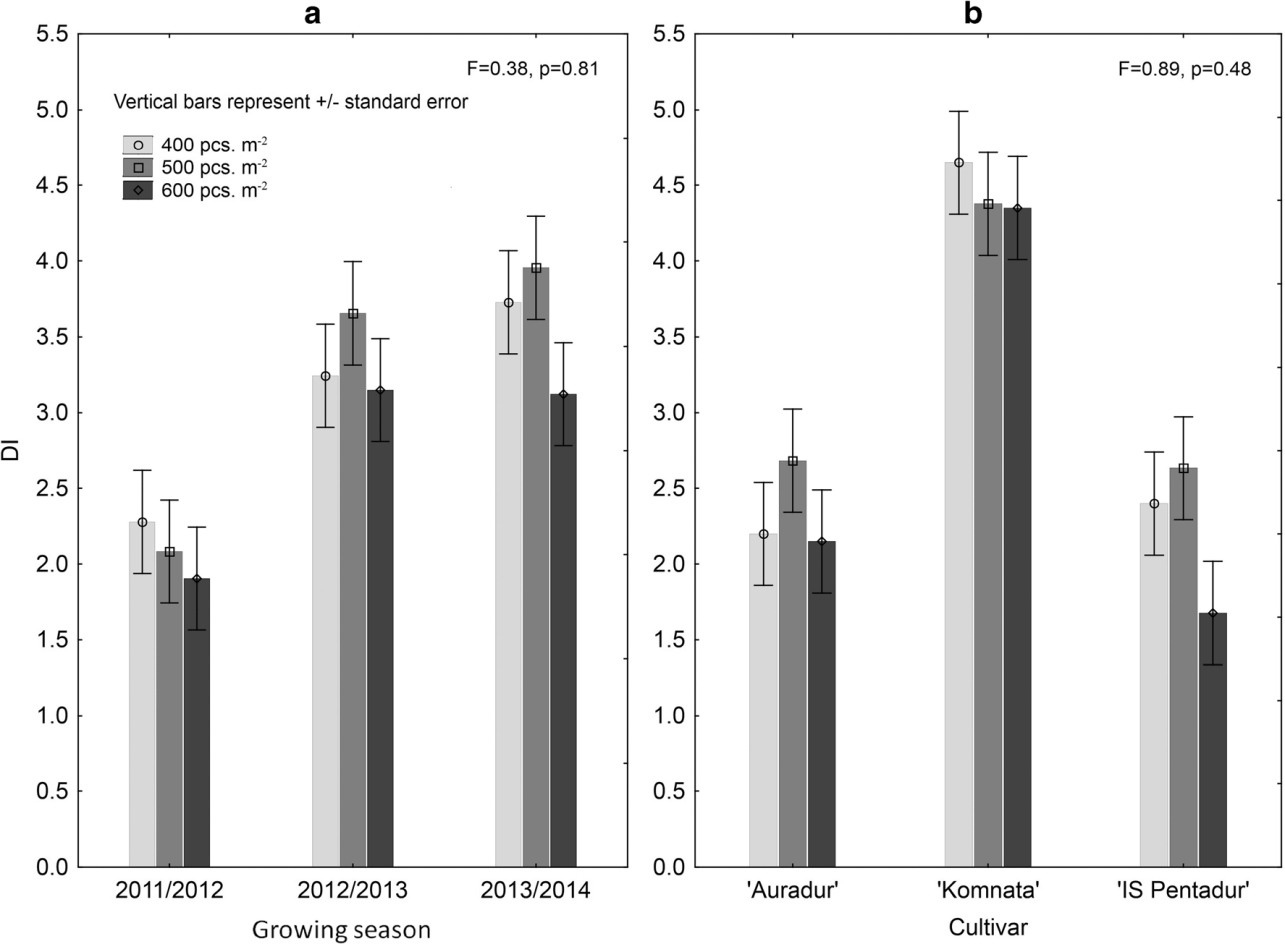
Sowing density interaction with year (**a**) and cultivar (**b**) on the FHB incidence in three durum wheat cultivars (400, 500, and 600 seeds per square meter were used). The differences between densities were not significant

The highest sowing density (600 per square meter) resulted in lower FHB incidence in all cultivars and across the three seasons; however, the differences between the densities (400, 500, and 600 seeds per square meter) were statistically not significant (Fig. 5). The cultivar’s reaction on the sowing density was different, as the most susceptible cv. Komnata displayed no reaction to the increased sowing density, while less susceptible cultivars (Auradur and IS Pentadur) showed the highest FHB incidence at moderate density (500 grains per square meter), particularly in 2012/2013 and 2013/2014 seasons (Fig. 5).

Sowing densities had no effect on the number of isolates obtained from samples analyzed; however, the delayed sowing date had positive impact on the number of isolated fungi: 15 isolates came from samples sown at delayed date in 2011/2012 season and 10 at optimal; 55 isolates were obtained for the samples sown at delayed date in 2012/2013 and 45 at the optimal, respectively. In 2013/2014 season, 53 isolates came from the samples sown at the delayed date and 33 from the samples sown at optimal date (Table 4). Nevertheless, no correlation was found between the number of isolates and mycotoxins measured for respective samples (Table 4).

### Mycotoxin accumulation

Komnata cultivar exhibited the highest correlation between the FHB level and mycotoxin contamination (Table S1). Analysis of variance showed that only “cultivar” and “year” were significant factors (Table 3). The highest FHB incidence on susceptible cv. Komnata (Fig. 2) was reflected by the number of *Fusarium* isolates and elevated mycotoxin content in the grain of this cultivar across all three seasons, particularly concerning deoxynivalenol (DON) concentrations (Tables 3 and 4). Moreover, cv. Komnata contained the greatest amounts of ZON and MON in 2013/2014 season and MON amounts in 2012/2013 season.

**Table 3:** Mean squares from four-way analysis of variance for observed disease symptoms (DI FHB) on winter durum wheat and mycotoxin contents (d.f. degrees of freedom, DON deoxynivalenol, ZON zearalenone, MON moniliformin, NIV nivalenol)

Source of variation	d.f.	DI FHB	DON	ZON	MON	NIV
Blocks	2	2.99	101,930	6.1	35.1	2.80
Sowing date (S)	1	3.38	23,546,358**	12,162.3**	57,936.1***	185.86
Residual 1	2	1.13	43,296	19.6	15.2	16.95
Cultivar (C)	2	84.80***	101,250,556***	924.8***	240,877.9***	25,272.02***
S × C	2	4.50*	814,259**	4520.9***	4184.1***	185.86***
Residual 2	8	0.73	57,807	43.2	196.6	9.87**
Sowing density (D)	2	3.65	770,619**	2525.0***	1539.0***	435.47***
S × D	2	1.01	218,367	4113.3***	11,493.2***	42.65***
C × D	4	1.44	2,376,267***	5305.9***	11,912.5***	435.47***
S × C × D	4	1.26	390,103**	5213.7***	3728.9***	42.65***
Residual 3	24	1.61	85,706	73.5	127.8	2.04
Year (Y)	2	35.49***	521,095,044***	101,804.3***	922,516.0***	25,272.02***
Y × S	2	6.22	7,126,441***	16,304.8***	47,274.9***	185.86***
Y × C	4	65.47***	32,423,647***	524.8***	194,090.4***	25,272.02***
Y × D	4	0.81	507,481***	4713.3***	2140.2***	435.47***
Y × S × C	4	2.45	3,626,389***	3796.0***	5454.0***	185.86***
Y × S × D	4	1.08	1,061,907***	6437.6***	13,184.1***	42.65***
Y × C × D	8	0.82	2,613,868***	4084.8***	12,554.0***	435.47***
Y × S × C × D	8	0.25	1,143,220***	4234.4***	4302.1***	42.65***
Residual 4	72	2.08	76,462	61.1	123.3	4.65

***P value below 0.05; *P* value below 0.01; ***P value below 0.001

**Table 4:** Deoxynivalenol (DON), zearalenone (ZON), moniliformin (MON), and nivalenol (NIV) present [in ng g^−1^] in grain samples of three durum wheat cultivars (Auradur, Komnata, IS Pentadur) harvested in 2011/2012–2013/2014 seasons, in following variants 400, 500, and 600 seeds per square meter. Total numbers of *Fusarium* strains isolated from individual samples were also given. MON was not detected in any of the samples in 2011/2012 season, and NIV was not detected in any of the samples in 2011/2012 and 2012/2013 season

Sample	DON	ZON	DON	ZON	MON	DON	ZON	MON	NIV
	2011/2012		2012/2013			2013/2014			
Optimal sowing date									
Auradur 400	nd	26.8 ± 5.3	4318.4 ± 567.2	163.0 ± 21.3	12.5 ± 2.3	526.5 ± 82.6	nd	140.7 ± 25.1	nd
Auradur 500	353.5 ± 125.7	1.1 ± 0.6	4425.0 ± 615.7	88.5 ± 25.0	12.8 ± 4.7	424.1 ± 57.9	nd	142.0 ± 40.6	nd
Auradur 600	253.9 ± 75.6	26.6 ± 8.4	3012.3 ± 29.8	41.1 ± 9.3	14.6 ± 5.4	533.2 ± 70.3	3.9 ± 0.7	137.8 ± 37.3	nd
Komnata 400	589.8 ± 69.3	6.0 ± 1.7	7565.1 ± 993.1	67.8 ± 10.5	31.9 ± 6.8	2306.7 ± 371.5	6.1 ± 0.9	525.6 ± 60.7	127.4 ± 26.1
Komnata 500	677.9 ± 112.0	3.1 ± 0.9	8637.6 ± 1402.5	114.7 ± 12.9	40.1 ± 9.0	1086.4 ± 99.8	5.2 ± 0.6	392.2 ± 35.4	88.1 ± 30.4
Komnata 600	893.9 ± 120.3	24.4 ± 7.2	10,879.6 ± 2417.6	106.6 ± 20.4	45.1 ± 9.5	1239.0 ± 201.4	10.2 ± 1.1	171.7 ± 51.6	92.7 ± 21.8
IS Pentadur 400	nd	nd	3528.4 ± 411.9	73.4 ± 80.5	9.1 ± 1.9	595.9 ± 93.5	4.4 ± 0.6	64.4 ± 10.9	nd
IS Pentadur 500	nd	2.0 ± 0.5	2767.0 ± 2005.8	307.3 ± 33.4	11.6 ± 2.5	851.9 ± 71.3	nd	40.2 ± 17.5	nd
IS Pentadur 600	nd	nd	3651.8 ± 417.3	46.8 ± 2.9	12.6 ± 5.0	1398.7 ± 154.7	10.4 ± 1.3	48.4 ± 16.3	nd
Dealyed sowing date									
Auradur 400	879.6 ± 93.7	4.0 ± 0.6	4711.1 ± 366.8	25.8 ± 3.6	32.8 ± 4.7	669.2 ± 52.6	3.2 ± 0.2	88.4 ± 36.8	nd
Auradur 500	nd	6.4 ± 0.9	5613.7 ± 629.7	41.1 ± 3.8	22.9 ± 5.9	743.9 ± 85.7	13.9 ± 1.5	195.8 ± 53.1	nd
Auradur 600	nd	5.2 ± 0.5	5012.6 ± 1035.8	90.4 ± 8.5	27.5 ± 2.0	541.8 ± 70.3	18.9 ± 5.5	317.9 ± 41.6	nd
Komnata 400	523.9 ± 82.5	15.9 ± 2.7	8724.8 ± 712.5	48.0 ± 5.2	46.3 ± 13.7	3307.8 ± 411.9	17.1 ± 5.9	521.5 ± 80.3	155.4 ± 37.9
Komnata 500	nd	2.4 ± 0.7	10,095.8 ± 1245.0	95.4 ± 11.0	37.1 ± 2.5	3358.8 ± 303.4	21.0 ± 2.7	579.8 ± 44.6	117.2 ± 44.3
Komnata 600	1030.2 ± 205.9	18.6 ± 2.6	10,740.9 ± 953.6	106.3 ± 9.8	42.4 ± 9.0	3988.6 ± 288.7	20.9 ± 8.0	500.5 ± 73.8	93.5 ± 10.8
IS Pentadur 400	nd	2.0 ± 0.5	6484.6 ± 711.2	40.6 ± 3.9	16.9 ± 3.2	717.0 ± 90.5	10.8 ± 3.4	160.2 ± 21.5	nd
IS Pentadur 500	487.0 ± 72.8	8.3 ± 1.9	6220.8 ± 894.1	30.2 ± 2.5	10.5 ± 1.7	825.2 ± 112.9	3.6 ± 0.5	125.5 ± 19.4	nd
IS Pentadur 600	nd	nd	4398.2 ± 563.8	189.0 ± 20.3	21.0 ± 7.3	2030.6 ± 185.3	2.8 ± 0.6	127.8 ± 36.0	nd

*nd* not detected

NIV was identified in the samples of cv. Komnata only, from which both DON and NIV chemotypes of *F. graminearum* were isolated during three seasons studied; however, those to be confirmed as NIV chemotype using chemotype-specific PCR marker were isolated in the last season only (results not shown).

## Discussion

FHB depends strongly on the environmental and weather conditions, which vary often between the seasons. Significant differences in FHB development and severity were observed during 2012–2014 seasons among the three cultivars tested. Low water content during the 2011/2012 season was reflected by just few *Fusarium* strains isolated, as well as by low mycotoxin contamination of the grain. Komnata was the most susceptible cultivar to the disease progress and mycotoxin accumulation through all three seasons, while cvs. Auradur and IS Pentadur were less susceptible. Studies conducted in various climatic conditions have proven a strong correlation between FHB epidemics and favorable temperatures and high humidity before and during flowering (Klem et al. 2007; Prandini et al. 2009; Shah et al. 2013; De Wolf et al. 2003). No significant host preference was observed, as similar *Fusarium* populations were found on common wheat in the area of Poland, except for *F. culmorum*, the most frequent species on common wheat, found on durum wheat only occasionally (Chełkowski et al. 2012; Wiśniewska et al. 2014).

Genetic resistance is a key feature in preventing the FHB epidemics, mycotoxin contamination (Bai and Shaner 2004), and selection of breeding materials towards disease-resistant genotypes. However, increased resistance to FHB seems to limit the occurrence of all pathogens of the complex (Fig. 3, Table 4). The genetic basis underlying this increased resistance has not yet been fully understood, and it could be hypothesized that some components of the possible host specificity have evolved in pathogen populations. Namely, *F. graminearum*, one of the main pathogens of maize, was not isolated at high frequencies lately (Czembor et al. 2015), though it was the second most abundant pathogen in the present study, proving that the inoculum source was present in the fields.

The southern part of Poland is the only area of the country where durum wheat is cultivated; therefore, selection of materials for FHB resilience can be more difficult than for other crops. One of the possible explanations for the differences in FHB susceptibility is that the cultivars bred in Austria and Slovakia have higher resistance levels than cultivars from Poland. This hypothesis would require extensive studies of durum wheat cultivars from respective countries to be verified. Interestingly, *F. avenaceum*, a species more typical for cooler climates (Stępień et al. 2013), was also spotted in the southern part of the Europe but mostly on common wheat (Covarelli et al. 2015). FHB susceptibility of wheat genotypes depends greatly on weather conditions promoting infection, which was confirmed in multi-year study on nearly 100 cereal genotypes (Landschoot et al. 2012).

No clear differences were observed in *Fusarium* species composition among cultivars tested; however, individual FHB-related species occurred at various frequencies, and some of them, namely *F. subglutinans*, *F. proliferatum*, and *F. verticillioides*, were found exclusively in the 2011/2012 season. Moreover, low number of strains isolated from the grains was reflected by the low levels of all mycotoxins quantified (Table 3). Differences in FHB susceptibility observed for cvs. sown at optimal date were reduced at the delayed sowing date with the exception of cv. Auradur in 2013/2014 season, which was severely diseased when sown on delayed date.

Increased sowing density usually positively correlates with FHB incidence due to amount of moisture kept between the plants; however, in the present study, medium sowing density (500 seeds per square meter) resulted in higher infection level (Fig. 5).

The highest level of FHB susceptibility expressed by cv. Komnata was confirmed by the grain contamination with DON, ZON, MON, and NIV. These mycotoxins were found also in samples, from which corresponding producers were not isolated (Table 4). It was particularly visible in the first season of experiments, when no pathogens were present in the samples tested. It is possibly due to low moisture content during ripening of the grain, which dramatically lowers the viability of fungi. Relationship between the disease index and mycotoxin contamination of the grain has been studied for many years, and the highest positive correlations have been reported for DON content (Bai et al. 2001; Khatibi et al. 2012; Paul et al. 2006). However, the opposite results have also been published (Ji et al. 2015; Liu et al. 1997; Mesterhàzy et al. 1999).

It can be concluded that the correlation between FHB severity and mycotoxin accumulation is mainly related to the cultivar used and specific weather conditions, being the highest in the seasons with high disease indices. Furthermore, the level of FHB correlated well with mycotoxins present in the grain (e.g., DON, MON, and NIV) in the present study, but no similar correlation was observed for ZON. In the case of the most susceptible cultivar (Komnata), this was valid for all mycotoxins analyzed. It can be hypothesized that the environmental conditions present in the Southern Europe are more suitable for selection and breeding of less susceptible materials than those bred in Poland.

## Electronic supplementary material

ESM 1 (PDF 161 kb)

## References

[CR1] Abad A, Lloveras J, Michelena A (2004). Nitrogen fertilization and foliar urea effects on durum wheat yield and quality and on residual soil nitrate in irrigated Mediterranean conditions. Field Crops Res.

[CR2] Bai GH, Shaner G (2004). Management and resistance in wheat and barley to *Fusarium* head blight. Annu Rev Phytopathol.

[CR3] Bai GH, Plattner R, Desjardins A, Kolb F (2001). Resistance to *Fusarium* head blight and deoxynivalenol accumulation in wheat. Plant Breed.

[CR4] Blandino M, Pilati A, Reyneri A (2009). Effect of foliar treatments to durum wheat on flag leaf senescence, grain yield, quality and deoxynivalenol contamination in North Italy. Field Crops Res.

[CR5] Chełkowski J, Gromadzka K, Stępień Ł, Lenc L, Kostecki M, Berthiller F (2012). *Fusarium* species, zearalenone and deoxynivalenol content in preharvest scabby wheat heads from Poland. World Mycotox J.

[CR6] Covarelli L, Beccari G, Prodi A, Generotti S, Etruschi F, Juan C, Ferrer E, Mañes J (2015). *Fusarium* species, chemotype characterisation and trichothecene contamination of durum and soft wheat in an area of central Italy. J Sci Food Agric.

[CR7] Czembor E, Stępień Ł, Waśkiewicz A (2015). The impact of environmental factors on *Fusarium* species and associated mycotoxins in maize grain grown in Poland. PLoS One.

[CR8] De Wolf ED, Madden LV, Lipps PE (2003). Risk assessment models for wheat *Fusarium* head blight epidemics based on within-season weather data. Phytopathology.

[CR9] Gana Y, Liang C, Wang X, McConkey B (2011). Lowering carbon footprint of durum wheat by diversifying cropping systems. Field Crops Res.

[CR10] Garcia del Moral LF, Rharrabti Y, Villegas D, Royo C (2003). Evaluation of grain yield and its components in durum wheat under Mediterranean conditions: an ontogenic approach. Agron J.

[CR11] Hossard L, Philibert A, Bertrand M, Colnenne-David C, Debaeke P, Munier-Jolain N, Jeuffroy MH, Richard G, Makowski D (2014). Effects of halving pesticide use on wheat production. Sci Rep.

[CR12] Janeczko A, Gruszka D, Pociecha E, Dziurka M, Filek M, Jurczyk B, Kalaji HM, Kocurek M, Waligórski P (2016). Physiological and biochemical characterisation of watered and drought-stressed barley mutants in the HvDWARF gene encoding C6-oxidase involved in brassinosteroid biosynthesis. Plant Physiol Biochem.

[CR13] Ji F, Wu J, Zhao H, Xu J, Shi J (2015). Relationship of deoxynivalenol content in grain, chaff, and straw with *Fusarium* head blight severity in wheat varieties with various levels of resistance. Toxins.

[CR14] Khatibi PA, Berger G, Liu S, Brooks WS, Griffey CA, Schmale DG (2012). Resistance to *Fusarium* head blight and deoxynivalenol accumulation in Virginia barley. Plant Dis.

[CR15] Klem K, Vànová M, Hajslová J, Lancová K, Sehnalová M (2007). A neural network model for prediction of deoxynivalenol content in wheat grain based on weather data and preceding crop. Plant Soil Environ.

[CR16] Labuschagne MT, Elago O, Koen E (2009). The influence of temperature extremes on some quality and starch characteristics in bread, biscuit and durum wheat. J Cereal Sci.

[CR17] Landschoot S, Waegeman W, Audenaert K, Vandepitte J, Baetens J, De Baets B, Haesaert G (2012). An empirical analysis of explanatory variables affecting *Fusarium* head blight infection and deoxynivalenol content in wheat. J Plant Pathol.

[CR18] Leslie JF, Summerell BA (2006). The fusarium laboratory manual.

[CR19] Liu W, Langseth W, Skinnes H, Elen ON, Sundheim L (1997). Comparison of visual head blight ratings, seed infection levels, and deoxynivalenol production for assessment of resistance in cereals inoculated with *Fusarium culmorum*. Eur J Plant Pathol.

[CR20] Lori GA, Sisterna MN, Haidukowski M, Rizzo I (2003). *Fusarium graminearum* and deoxynivalenol contamination in the durum wheat area of Argentina. Microbiol Res.

[CR21] Mesterhàzy A, Bartók T, Mirocha CG, Komoróczy R (1999). Nature of wheat resistance to *Fusarium* head blight and the role of deoxynivalenol for breeding. Plant Breed.

[CR22] Paul PA, Lipps PE, Madden LV (2006). Meta-analysis of regression coefficients for the relationship between Fusarium head blight and deoxynivalenol content of wheat. Phytopathology.

[CR23] Pierre JG, Regnault Y (1982). Contribution `a la mise au point d’une methode de plein champ destinee a mesurer la sensibilite des varietes de colza au phoma. Informations Techniques du CETIOM.

[CR24] Prandini A, Sigolo S, Filippi L, Battilani P, Piva G (2009). Review of predictive models for *Fusarium* head blight and related mycotoxin contamination in wheat. Food Chem Toxicol.

[CR25] Royo C, Aparicio N, Blanco R, Villegas D (2004). Leaf and green area development of durum wheat genotypes grown under Mediterranean conditions. Eur J Agr.

[CR26] Royo C, Villegas D, Rharrabti Y, Blanco R, Martos V, Garcia del Moral LF (2006). Grain growth and yield formation of durum wheat grown at contrasting latitudes and water regimes in a Mediterranean environment. Cereal Res Commun.

[CR27] Royo C, Nazco R, Villegas D (2014). The climate of the zone of origin of Mediterranean durum wheat (*Triticum durum* Desf.) landraces affects their agronomic performance. Genet Res Crop Evol.

[CR28] Shah DA, Molineros JE, Paul PA, Willyerd KT, Madden LV, De Wolf ED (2013). Predicting *Fusarium* head blight epidemics with weather-driven pre- and post-anthesis logistic regression models. Phytopathology.

[CR29] Stępień Ł, Chełkowski J, Wenzel G, Mohler V (2004). Combined use of linked markers for genotyping the *Pm1* locus in common wheat. Cell Mol Biol Lett.

[CR30] Stępień Ł, Gromadzka K, Chełkowski J (2012). Polymorphism of mycotoxin biosynthetic genes among *Fusarium equiseti* isolates from Italy and Poland. J Appl Genet.

[CR31] Stępień Ł, Jestoi M, Chełkowski J (2013). Cyclic hexadepsipeptides in wheat field samples and *esyn1* gene divergence among enniatin producing *Fusarium avenaceum* strains. World Mycotox J.

[CR32] Stępień Ł, Waśkiewicz A, Urbaniak M (2016). Wildly growing asparagus (*Asparagus officinalis* L.) hosts pathogenic *Fusarium* species and accumulates their mycotoxins. Microbial Ecol.

[CR33] Tomczak M, Wiśniewska H, Stępień Ł, Kostecki M, Chełkowski J, Goliński P (2002). Deoxynivalenol, nivalenol and moniliformin in wheat samples with head blight (scab) symptoms in Poland (1998-2000). Eur J Plant Pathol.

[CR34] Wiśniewska H, Stępień Ł, Waśkiewicz A, Beszterda M, Góral T, Belter J (2014). Toxigenic *Fusarium* species infecting wheat heads in Poland. Central Eur J Biol.

