# Supplementary material for: Fusarium head blight incidence and mycotoxin accumulation in three durum wheat cultivars in relation to sowing date and density
Source: Naturwissenschaften. 2017 Dec 5;105(1):2. doi: 10.1007/s00114-017-1528-7 (PMC5717115; doi:10.1007/s00114-017-1528-7)
Supplement: Supplementary file 1 — (PDF 161 kb) [file 114_2017_1528_MOESM1_ESM.pdf]

Table S1

The coefficients of linear correlation between symptoms of Fusarium Head Blight (FHB) and mycotoxin contents measured for cultivar Komnata.

Mycotoxins	Growth season 2013/2014	Cultivar Komnata	For all factors tested
Deoxynivalenol	0.76**	-0.39	0.24
Zearalenone	0.61**	-0.50*	0.13
Moniliformin	0.89**	0.92**	0.69**
Nivalenol	0.94**	0.93**	0.80**

* P value below 0.05

** P value below 0.01
